# Race and Gender on the Mortality of Giant Cell Arteritis in Hospitalized Patients: A 15-Year National Inpatient Study

**DOI:** 10.7759/cureus.46165

**Published:** 2023-09-28

**Authors:** Abiodun B Idowu, Pushti Khandwala, Irene J Tan

**Affiliations:** 1 Internal Medicine, Einstein Medical Center Philadelphia, Philadelphia, USA; 2 Rheumatology, Einstein Medical Center Philadelphia, Philadelphia, USA

**Keywords:** mortality, ethnicity, race, giant cell arteritis, gender

## Abstract

Background: Critical appraisal of mortality in giant cell arteritis (GCA) through a racial lens is imperative as gender and racial disparities remain a global healthcare concern.

Objective: To analyze the impact of race and gender on the mortality of GCA in United States (US)-hospitalized patients.

Methods: In this retrospective cohort study, the National Inpatient Sample (NIS) database from January 2003 to December 2018 was searched to identify all patients aged >18 years hospitalized with giant cell arteritis. Patients’ baseline characteristics were summarized using descriptive statistics. Inferential statistics were done for categorical and continuous variables. Multivariate logistic regression, adjusting for patient and hospital-level cofounders was performed to find an association between race and outcomes of interest.

Results: Over the 15-year study period, a total of 8,352 patients (72.7% White, 14.5% Black or African American, 7.6% Hispanic, 2.2% Asian, 0.4% Alaska Native, and 2.6% under-represented populations) were hospitalized for GCA. The mean age at diagnosis was 73.6 ± 0.12 years. Women represented 71.9% of GCA patients and had a lower risk of mortality (OR 0.463, 95% CI: 0.235 - 0.912, p <0.05). Patients with GCA were hospitalized for an average of 4.64 days ± 0.04 days and 0.55% died. The mortality rate was lowest in Black or African American (0.1%) populations and highest among Alaska Native patients (8%). Mortality was 68% lower in those who had temporal artery biopsy (OR 0.32, 95% CI: 0.16-0.64, p <0.05).

Conclusion: GCA disproportionally affected female patients, but mortality was higher in male patients. Alaska Native patients had the least number of hospitalizations but the highest in-hospital mortality rate. Black or African Americans had the lowest mortality rate.

## Introduction

Giant cell arteritis (GCA), previously referred to as temporal arteritis, is the most commonly occurring systemic vasculitis in adults older than 50 [1]. The etiology of this granulomatous vasculitis of large- and medium-sized arteries is still unknown [2]. However, when not promptly diagnosed and treated appropriately, it can lead to irreversible sequelae, such as blindness, ischemic cardiovascular and cerebrovascular events, and poor quality of life [3].

Race and ethnicity are significant determinants of outcomes in many medical disorders [4,5]. Patients who are White, especially those with origin from either Northern Europe or North America, have a higher prevalence of GCA [6] while the reported prevalence is significantly lower in Hispanic, Asian, and Black or African Americans [7-8]. These reports provide valuable data on the global incidence of GCA. However, the applicability of these data in the United States (US) population is unclear given the extensive racial diversity. We set out to determine the prevalence of GCA across different races and genders, as well as the impact of race on mortality outcomes in US-hospitalized patients with objective nationally representative data. The current study was undertaken using a 15-year hospitalized patients’ database in the US. 

## Materials and methods

Database description

Data for this study was obtained from the 2003-18 Nationwide Inpatient Sample (NIS). The NIS of the Health Care Utilization Project (HCUP) is the largest publicly available (comprises a 20% stratified random sample of all US hospital discharges), de-identified, discharge-level inpatient database in the United States sponsored by the Agency for Healthcare Research and Quality [9]. The NIS is an annual sample of hospital discharges that provides national estimates of the characteristics of the patients, diagnoses, and hospital-based procedures performed in US acute-care hospitals. All hospital discharges from the sample are weighed to ensure that they are nationally representative. 

Study population

We identified all patients with a primary diagnosis of GCA by querying the database using the International Classification of Disease-Clinical Modification, 10th revision (ICD-10 CM) codes "M31.5" and "M31.6", and ICD, 9th revision (ICD-9 CM) code "446.5" (Appendix table).

Race and ethnicity description

Depending on each hospital’s policies, patients answer either open-ended questions or a fixed set of categories on their racial and or ethnic identity before issuance of a medical record number. In this study, the patient’s race or ethnicity was reported as the patient’s self-identified and reported into the HCUP database [9]. Herein, ‘Alaska Native’ refers to a person having origins in any of the original peoples of North and South America (including Central America), ‘Asian’ refers to a person having origins in any of the original peoples of the Far East, Southeast Asia, or the Indian subcontinent. ‘Black or African American’ refers to a person having origins in any of the black racial groups of Africa. ‘Hispanic’ refers to a person of Cuban, Mexican, Puerto Rican, South or Central American, or other Spanish culture or origin. ‘White’ refers to a person having origins in any of the original peoples of Europe, the Middle East, or North Africa. 

Statistical analysis

All data analyses were performed using SAS 9.4 (SAS Institute Inc, Cary, North Carolina). Results were presented as numbers and/or percentages. Categorical variables were compared using the Rao-Scott Chi-square test or Fisher’s exact test. Continuous variables compared using the Student t-test or analysis of variance (as appropriate). Multivariable logistic regression analysis was used to calculate adjusted odds ratios (aOR) for outcomes of interest. Analysis was adjusted for age, race, gender, hospital region, and patient comorbidities (chronic kidney disease, ischemic heart disease, diabetes mellitus, hypertension, smoking, obesity). All analyses were done at a 95% confidence interval (CI), and a 2-tailed p-value <0.05 was considered statistically significant. 

Ethical consideration

The NIS is a publicly available de-identified database; therefore, institutional review board approval was not required for our study.

## Results

**Figure 1:**
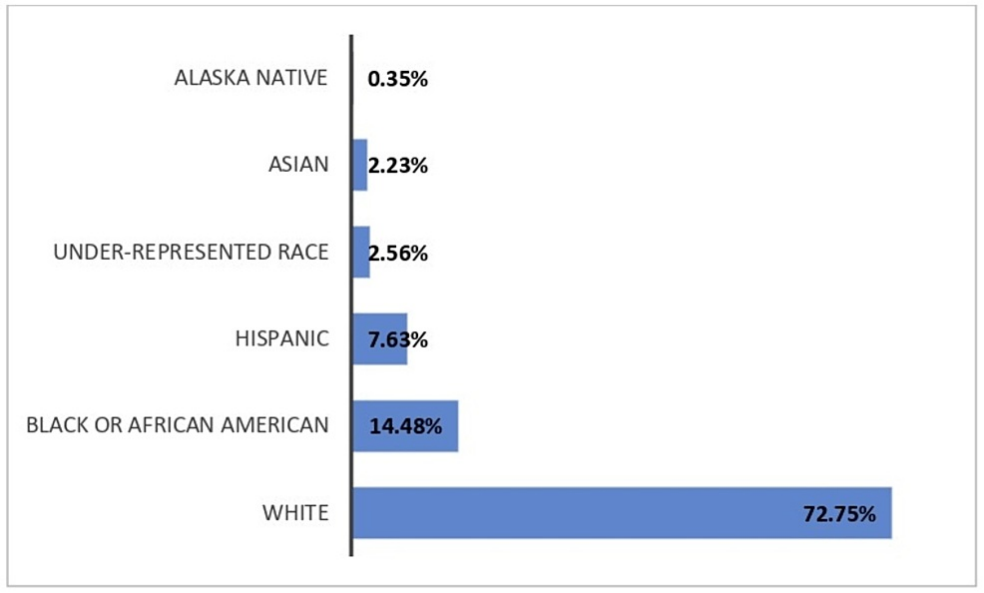
Racial distribution of patients hospitalized with GCA within the 15 years survey period

A total of 8,352 patients with GCA were admitted over the 15-year survey period. We found that 0.35% were Alaska Native, 2.23% Asian, 14.48% Black or African American, 7.63% Hispanic, 72.75% White, and 2.56% belonged to underrepresented populations (Figure 1).

The mean age of the patients was 73.64 years (65.06% were >70 years) and 71.86% were female. A total of 1.63% of the patients with GCA had bilateral blindness, 3.41% had unilateral blindness, 8% were obese, 12% had comorbid chronic kidney disease, 13% were smokers, 25% had concomitant history of ischemic heart disease, 31% had type 2 diabetes mellitus, and 57% had comorbid chronic hypertension (Table 1). 

**Table 1:** Patient’s demographics and comorbidities in association with temporal artery biopsy Total and temporal biopsy columns are presented as N (%). Age reported as mean ± SD. p <0.05 is considered statistically significant. ≠ indicates missing data (for eight patients)

	Total (%)	Temporal Artery Biopsy		p-value
		Yes (56.23%)	No (43.77%)	
Age in years (mean ± SD)	73.64 ± 0.12	73.43 ± 0.16	73.86 ± 0.19	0.084
Female	6000 (71.86)	3340 (55.67)	2660 (44.33)	0.101
Male	2349 (28.14)	1354 (57.64)	995 (42.36)	
Race ^≠^				0.217
Alaska Native	25 (0.35)	13 (52.00)	12 (48.00)	
Asian	161 (2.23)	93 (57.76)	68 (42.24)	
Black or African American	1047 (14.48)	571 (54.54)	476 (45.46)	
Hispanic	552 (7.63)	302 (54.71)	250(45.29)	
White	5260 (72.75)	2995 (56.94)	2265 (43.06)	
Underrepresented	185 (2.56)	118 (63.78)	67 (36.22)	
Co-morbidities				
Hypertension	4760 (56.99)	2709 (56.92)	2051 (43.09)	0.146
Type 2 Diabetes Mellitus	2567 (30.74)	1400 (54.54)	1167 (45.46)	0.038
Ischemic Heart Disease	2051 (24.56)	1114 (54.32)	937 (45.68)	0.045
Chronic Kidney Disease	1029 (12.32)	555 (53.94)	474 (46.06)	0.114
Smoking	1056 (12.64)	625 (59.19)	431 (40.81)	0.038
Obesity	635 (7.60)	358 (56.38)	277 (43.62)	0.936
Unilateral Blindness	285 (3.41)	154 (54.04)	131 (45.96)	0.448
Bilateral blindness	136 (1.63)	79 (58.09)	57 (41.91)	0.659

In terms of the hospital region within the US, 38.8% were hospitalized in the South, 24.56% in the Northeast, 22.02% in the Midwest, and 14.62% in the West. On average, patients with GCA were hospitalized for 4.64 days, incurred 34,142 US dollars in cost of hospitalization, and 0.55% died while hospitalized (Table 2). A total of 4,696 (56.23%) patients had temporal artery biopsy (TAB) done at the index admission. The biopsy rate was not significantly influenced by the patients' age, race, gender, or comorbidities (Table 1). TAB was performed significantly more in the hospitals in the Northeast region (59.34%) followed by those in the South (56.96%), Midwest (56.28%), and West (48.98%) (p<0.0001) (Table 2). The TAB procedure was not significantly associated with the insurance status of the patients. Individuals who had TAB stayed significantly longer in the hospital (5.02 ± 0.05 vs 4.12 ± 0.08, p<0.0001), had a higher cost of hospitalization (p<0.001) but had lower all-cause inpatient mortality (26% vs 74%, p<0.001) (Table 2).

**Table 2:** Hospital region, insurance, cost of hospitalization, length of stay and mortality in association with temporal artery biopsy status Total and temporal biopsy columns are presented as N (%). p <0.05 is considered statistically significant. ≠ indicates missing data (for eight patients)

	Total (%)	Temporal Artery Biopsy		p-value
		Yes (56.23%)	No (43.77%)	
Hospital Region				<0.0001
Northeast	2051 (24.56)	1217 (59.34)	834 (40.66)	
Midwest	1839 (22.02)	1035 (56.28)	804 (43.72)	
South	3241 (38.80)	1846 (56.96)	1395 (43.04)	
West	1221 (14.62)	598 (48.98)	623 (51.02)	
Patients’ Insurance^≠^				0.226
Medicare	6446 (77.25)	3604 (55.91)	2842 (44.09)	
Medicaid	434 (5.20)	233 (53.69)	201 (46.31)	
Private	1180 (14.14)	691 (58.56)	489 (41.44)	
Self-Pay	154 (1.85)	83 (53.90)	71 (46.10)	
Others	130 (1.56)	82 (63.08)	48 (36.92)	
Cost of Hospitalization in US Dollars (mean ± SD)	34142 ± 399.39	39686.9 ± 510.4	27272.3 ± 611.1	<0.0001
Length of stay in days (mean ± SD)	4.64 ± 0.04	5.02 ± 0.05	4.12 ± 0.08	<0.0001
All-cause mortality	46 (0.55)	12 (26.09)	34 (73.91)	<0.0001

A total of 46 of the 8352 patients died in-hospital (0.6%). Patients that died were older (79.48 ± 1.20 vs 73.58 ± 0.12, p=0.0004) and were mostly male patients (0.85% vs 0.43%, p =0.0205) (Table 3). The mortality rate was highest among Alaska Native patients (8%) and lowest (0.1%) among the Black or African American population. Longer hospitalization (14.02 ± 3.31 vs 4.57 ± 0.04, p<0.0001) and higher cost of hospitalization (99835.4 ± 21980 vs 33903.2 ± 377.8, p<0.0001) were seen in patients who died (Table 3). Temporal artery biopsy is associated with significantly lower odds of mortality by 68% (p<0.05) (Table 3).

**Table 3:** All-cause mortality among patients hospitalized with GCA p <0.05 was considered statistically significant.

	Died In-hospital		p-value
	Yes (%)	No (%)	
Age in years (mean ± SD)	79.48 ± 1.20	73.58 ± 0.12	0.0004
Female	0.43%	99.57%	0.0205
Male	0.85%	99.15%	
Race			<0.0001
White	0.53%	99.47%	
Black or African American	0.10%	99.90%	
Hispanic	0.36%	99.64%	
Asian	0.62%	99.38%	
Alaska Native	8.00%	92.00%	
Underrepresented	1.62%	98.38%	
Hospital region			0.7605
Northeast	0.49%	99.51%	
Midwest	0.43%	99.56%	
South	0.65%	99.35%	
West	0.57%	99.43%	
Cost of Hospitalization in US Dollars (mean ± SD)	99835.4 ± 21980	33903.2 ± 377.8	<0.0001
Length of Stay in days (mean ± SD)	14.02 ± 3.31	4.57 ± 0.04	<0.0001
Temporal Artery Biopsy	0.26%	99.74%	<0.0001

Female patients have 54% lower odds of mortality from the GCA hospitalization compared to males (p<0.05). Alaska Native patients were 27-fold more likely to die when hospitalized for GCA compared to White patients (p<0.05). Those who underwent temporal artery biopsy were 68% less likely to die compared to those who did not have temporal artery biopsy (p<0.05) (Table 4). 

**Table 4:** Multivariate regression analysis of In-hospital mortality outcome Analysis was adjusted for age, race, gender, hospital region, and patient comorbidities (chronic kidney disease, ischemic heart disease, diabetes mellitus, hypertension, smoking, obesity).

	Adjusted Odds Ratio	95% CI	p-value
Gender			
Male	reference		
Female	0.463	(0.235 - 0.912)	<0.05
Hospital region			
Northeast	reference		
Midwest	0.913	(0.417 - 2.739)	>0.05
South	1.493	(0.702 - 3.177)	>0.05
West	1.203	(0.459 - 3.150)	>0.05
Race			
Alaska Native	27.330	(5.513 - 135.486)	<0.05
Asian	1.172	(0.156 - 8.803)	>0.05
Black or African American	0.284	(0.039 - 2.079)	>0.05
Hispanic	0.951	(0.216 - 4.182)	>0.05
White	reference		
Underrepresented population	4.143	(1.234 - 13.814)	<0.05
Temporal Artery Biopsy	0.323	(0.162 - 0.643)	<0.05

## Discussion

Our study explores the gender and racial differences in the diagnosis and outcome of GCA for hospitalized patients in the US. We found that the diagnosis of GCA increases with age, with a significant rise after > 50 years of age. Our study's mean age of patients with a new diagnosis of GCA was 73.6 ± 0.12 years, consistent with reports of other earlier studies [10-13]. These results consistently showed that GCA has a predilection for the elderly, and this predisposition is consistent irrespective of the geographic location. Our study supports the entry criteria of age of more than 50 years as an absolute requirement for consideration of GCA diagnosis according to the most recent ACR/EULAR classification criteria in 2022 [14]. 

In terms of gender, GCA affected females more than male patients with a female-to-male ratio of 2.5:1; a slight difference in order of magnitude compared with data from India, where the cumulative female-to-male frequency based on 72 cases is estimated at 1.32:1 [15]. This contrasts with the 1:1.22 female-to-male affected rate in China based on a single-center retrospective study with 91 GCA patients [16]. The differences between the female-to-male prevalence rate of these studies and our study may be related to the type and setting of the study, and the sample size. A study similar to ours included 141 people of Aboriginal descent in North America and reported a female-to-male ratio of 2.4:1 [17]. It is yet unknown why GCA disproportionately affects females more than males. Like other auto-immune diseases, we hypothesize that hormonal and genetic differences may be contributory. 

Hospitalization for GCA is highest among patients who are White and lowest among patients who self-identified as Asian and Alaska Native (figure 1). Studies have shown that GCA is seven-fold lower among the African American population and 20 times less likely for Asians living in the US [18,19]. Differences in HLA-DR4 polymorphism have been implicated in GCA, and variation in the genetic expression of these HLA subtypes may account for racial differences [20]. 

Our study's temporal artery biopsy rate (TAB) was 56%. There was no significant association between biopsy rate and patients’ factors such as age, gender, race, comorbid conditions, insurance status, and visual impairment status. We opine that the relatively low biopsy rate is due partly to the evolving GCA classification criteria guideline which continued to undergo various iterations from 1990-2022. In the recent past, the diagnosis of GCA is traditionally based on the American College of Rheumatology (ACR) classification criteria. Though temporal artery biopsy was one of the scoring system's components, it is often done when the other components' score is equivocal [21]. The ACR/EULAR has recently released new criteria with 100% sensitivity and 94.9% specificity for diagnosing temporal artery biopsy-proven GCA [14]. Our study noted that the use of non-biopsy diagnosis is associated with reduced cost and length of hospitalization. However, those who had TAB had a 68% significant reduction in the odds of all-cause mortality. Further patient-level research would be needed to evaluate the impact of TAB on the inpatient mortality rate. 

Earlier studies have shown that GCA is the second leading cause of death in primary vasculitides in the US [22]. Nonetheless, the risk of GCA-related death stays lower as <1% of GCA hospitalized patients died in-hospital. The low mortality rate of 0.55% in our study is in alignment with the conclusions that patients with GCA are not at higher risk of mortality than the general population [23-25]. Our study showed a nearly two-fold increase in all-cause mortality in hospitalized male versus female GCA patients which is similar to a study in the United Kingdom [26]. In contrast, meta-analyses as well as a Danish population nationwide study showed that the risk of all-cause death in patients with GCA did not differ substantially by gender [23,27,28].

We found a striking inverse relationship between the prevalence of GCA among certain racial groups and the rate of all-cause inpatient mortality. GCA occurs less often among Alaska Native and Asian when compared to White patients. However, the inpatient mortality rate among those admitted for GCA is highest among the Alaska Native population and is least among Black or African Americans. Alaska Native patients were 27-fold more likely to die than White patients. This finding was similar to earlier country-level national mortality data from 1999-2019, which showed that the adjusted vasculitis-related mortality rate was higher in the Alaska Native population than in White patients and least in Black or African Americans [22]. The mortality rate was not significantly associated with insurance status, suggesting that the high mortality rate among the Alaska Native population may be more related to the multifaceted systemic inequalities in access to quality healthcare and not insurance status. A survey showed that compared to the other races, Alaska Native patients have similar healthcare coverage but a lower prevalence of having a personal healthcare provider [29]. A qualitative study on the barriers to quality healthcare found that high provider turnover, long travel time, and prolonged wait time at healthcare sites were some contextual barriers faced by elderly Native Americans and Alaska Native patients [30]. 

This current study has limitations inherent to any retrospective study. First, the diagnosis of GCA is not standardized and is completely dependent on physicians and institutional practices. In addition to the absence of diagnostic criteria for GCA, the guideline for the classification of GCA underwent modifications during the years examined in our study. This non-uniformity in making the diagnosis is an intrinsic selection bias. Second, the result of this study should be interpreted with the contextual understanding that race was self-reported in the database and not based on genetic determination or strict cut-off. Despite these limitations, a notable strength of this index study is that it is one of the few studies that used an extensive population database spanning over 15 years to report the impact of gender and race on the outcome of GCA.

## Conclusions

In the US, hospitalization for patients with giant cell arteritis was highest in Whites and least in the Alaska Native population. About two-thirds of patients with GCA were older than 70 years of age. The in-hospital mortality rate was highest in patients who identified as Alaska Native and lowest in the Black or African American population. Although GCA was more common in females, the mortality rate in males was higher. The use of temporal artery biopsy for the diagnosis of GCA was not significantly influenced by the patients' race or gender. Patients who underwent temporal artery biopsy had a significant reduction in the odds of all-cause in-hospital mortality.

## Appendices

Table 5 shows the ICD codes for the variables used in this study. 

**Table 5:** Supplementary table ICD codes for the variables used in this study

ICD 9 Codes	ICD 10 Codes	Diagnosis
369.0	H54.0	Bilateral Blindness
369.1, 369.6	H54.1, H54.4	Unilateral Blindness
Procedure code 38.21	Procedure code 03BS, 03BT	Temporal artery biopsy
401	I10	Hypertension
410, 411, 412, 413, 414	I20, I21, I22, I23, I24, I25	Ischemic heart disease
585	N18	Chronic Kidney disease
305.1, V15.82	Z72.0	Smoking
250.00, 250.02, 250.10, 250.12, 250.20, 250.22, 250.30, 250.32, 250.40, 250.42, 250.50, 250.52, 250.60, 250.62, 250.70, 250.72, 250.80, 250.82, 250.90, 250.92	E11	Diabetes mellitus type 2
278.00, 278.01, V85.3, V85.4	E66.0, E66.1, E66.8, E66.9 Z68.3, Z68.4	Obesity
